# Resolving Metabolic Heterogeneity in Experimental Models of the Tumor Microenvironment from a Stable Isotope Resolved Metabolomics Perspective

**DOI:** 10.3390/metabo10060249

**Published:** 2020-06-15

**Authors:** Teresa W. -M. Fan, Richard M. Higashi, Yelena Chernayavskaya, Andrew N. Lane

**Affiliations:** 1 Center for Environmental and Systems Biochemistry, Lexington, KY 40536, USA; rick.higashi@uky.edu (R.M.H.); ych354@uky.edu (Y.C.); 2 Department of Toxicology and Cancer Biology, University of Kentucky, Lexington, KY 40536, USA

**Keywords:** tumor microenvironment, 3D cultures, tissue slices, stable isotope resolved metabolomics

## Abstract

The tumor microenvironment (TME) comprises complex interactions of multiple cell types that determines cell behavior and metabolism such as nutrient competition and immune suppression. We discuss the various types of heterogeneity that exist in solid tumors, and the complications this invokes for studies of TME. As human subjects and in vivo model systems are complex and difficult to manipulate, simpler 3D model systems that are compatible with flexible experimental control are necessary for studying metabolic regulation in TME. Stable Isotope Resolved Metabolomics (SIRM) is a valuable tool for tracing metabolic networks in complex systems, but at present does not directly address heterogeneous metabolism at the individual cell level. We compare the advantages and disadvantages of different model systems for SIRM experiments, with a focus on lung cancer cells, their interactions with macrophages and T cells, and their response to modulators in the immune microenvironment. We describe the experimental set up, illustrate results from 3D cultures and co-cultures of lung cancer cells with human macrophages, and outline strategies to address the heterogeneous TME.

## 1. Introduction

### 1.1. Heterogeneity in the Tumor Microenvironment (TME)

Most tissues and solid tumors are highly heterogeneous at the molecular, cellular, and regional levels, as well as across entire organ. Thus, heterogeneity is the rule rather than the exception. Table 1 summarizes some of the known types of heterogeneity in solid tumors [1,2,3,4,5,6,7,8,9,10,11,12,13].

Heterogeneity imposes many problems for detailed analysis of the molecular and cellular behavior of solid tumors, partly because of technical limitations. This is critical to the fundamental understanding of tumor biology and the design of therapeutic strategies. Nevertheless, considerable progress is being made via single cell analyses of genome, transcriptome [13,21,22,40,41,42,43,44,45,46,47,48,49], proteome [50,51,52], and metabolome [8,52,53,54,55,56,57]. Although spatially resolved single cell metabolism has long been studied by live cell microscopy, the number of metabolites that can be detected and quantified is very limited [58,59,60,61]. The more recent single cell metabolomics development can capture more metabolites, but it is limited to those at high abundance while there are important issues on quantitation and reproducibility yet to be resolved [55]. Moreover, a major issue in single-cell analysis is how to preserve biochemical integrity, cell–cell interactions, and spatial configuration during measurement. These aspects of cell and tissue architecture are essential to our understanding of cell behavior in the heterogeneous environment. For example, cells behavior differs according to direct homo and heterotopic contacts versus interactions via diffusing molecules or vesicles [62,63].

**Figure 1:**
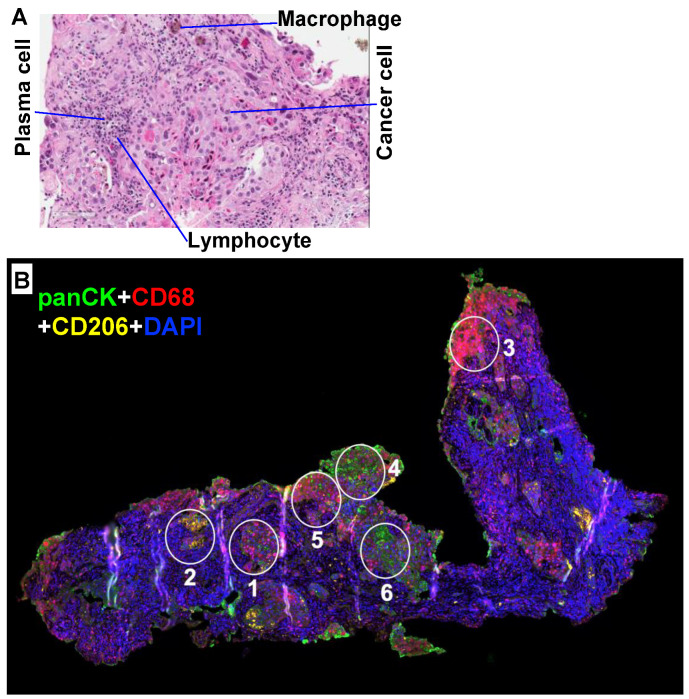
Heterogenous cellularity in organotypic tissue cultures (OTC) of non-small cell lung cancer (NSCLC) patient tumor tissues. The freshly resected NSCLC patient tumor tissues were thinly sliced and incubated in Dulbecco’s Modified Eagle’s Medium (DMEM) medium as OTC for 24 h at 37 °C/5% CO_2_ before fixing in 4% buffered formalin, embedded in paraffin block, and sectioned into 4 µm slices for H&E staining in (**A**) and IF staining for cancer cells (panCytokeratin or panCK), CD8 (cytotoxic T cells), and CD68 (Mφ) in (**B**). Highly heterogeneous distribution of cancer cells and various immune cell types is evident. White circles (200 μm diameter) in (**B**) defined the regions of interest (ROI) for the ROI-specific analysis (DSP) of 58 different protein markers that reflect immune functions. Each ROI was enriched in CD8 T cells (**3**), Mφ (**2**), and cancer cells (**4**, **6**), or contained a mixture the three cell types (**1**, **5**).

Figure 1A shows an example of heterogeneous cellular distribution in non-small cell lung cancer (NSCLC) tissues visualized with hematoxylin and eosin (H&E) stain. Different cell types including cancer cell and immune cells and their distributions are assessed morphologically. With the recent development of Digital Spatial Profiling (DSP), multiplexing bar-coded antibodies or oligonucleotide probes each for a different protein or mRNA target is employed to obtain the spatial distribution of multiple targets in tissues at single cell type resolution [64,65,66] (e.g., Figure 1B). What is clear from Figure 1 is the highly heterogeneous distribution of different cell types. As cancer cells are found in different microenvironments, their biochemical properties are likely to vary across the tissue field. Indeed, scRNAseq profiles reveal heterogeneity in mRNA expression not only among different cell types but also within each cell type [46,67,68,69]. However, mRNA levels do not always translate to protein levels and metabolic functions [45,70,71,72,73], which therefore needs verification. Furthermore, the TME is strongly influenced by the vasculature, which can limit the blood flow and thus O_2_/nutrient supply as well as waste product removal [18,74,75], and by the extracellular matrix (ECM) deposited by cancer-associated fibroblasts (CAF) [67,76,77,78]. Together, these factors not only dictate the availability of nutrients and competition among cell types for these nutrients but can also alter the microenvironment to favor cancer cell growth [79].

### 1.2. Stable Isotope Resolved Metabolomics (SIRM)

Metabolic profiling of tissues and biofluids including blood plasma [80,81,82,83], urine [84,85,86], and cerebrospinal fluid (CSF) [86,87,88,89] using NMR and MS in the past decade has been shown to be very valuable for reporting disease states and drug responses. This is due to metabolic reprogramming in response to disease development, particularly for cancer. In fact, metabolic reprogramming is a hallmark of human cancer that drives cancer development and progression [28]. However, profiles of steady-state metabolite levels determined by NMR and/or MS cannot resolve intersecting and compartmentalized metabolic networks, as given metabolite levels are influenced by multiple factors such as rates of synthesis and/or degradation, multiple inputs/outputs, and exchanges across compartments. However, the complexity of metabolic networks can be resolved with the use of stable isotope tracers coupled with metabolomic analysis, which we termed Stable Isotope-Resolved Metabolomics or SIRM [90]. SIRM enables individual atoms in tracers such as ^13^C_6_-glucose to be tracked through metabolic transformations so that intersecting and compartmentalized metabolic networks can be rigorously reconstructed without the ambiguities known to metabolite profiling-based pathway analysis described above. Stable isotopes especially ^13^C, ^15^N, and ^2^H (D) are fully biologically compatible and isotopically enriched substrates can be administered with no or minimal biological effects. Stable isotope tracing of metabolic pathways has been demonstrated in a wide variety of biological systems [32,91,92,93,94,95,96,97] for the reconstruction of metabolic networks [98,99,100] and also, in some cases, mapping of metabolic flux [77,90,91,101,102,103,104,105,106].

Currently, single tracers predominate in SIRM studies. Despite the technical challenges, there are substantial advantages in developing multiplexed SIRM (mSIRM) approaches where multiple precursors containing different tracer atoms such as ^13^C, ^15^N, and D are used together. These include large expansion of metabolic network coverage without interferences from sample batch variations while greatly reducing sample requirement; the latter is crucial to studies with very limited patient-derived (PD) materials such as PD organotypic tissue culture (OTC) or organoid (PDO) studies. We have begun such development, as described in Fan et al. [107].

## 2. Advantages and Disadvantages of Different Model Systems 

There are many model systems available for studying basic biological mechanisms and preclinical assessment of drug efficacy; each has its own advantages and disadvantages and thus should be tailored to the specific problem [108].

### 2.1. 2D Cell Models

Two-dimensional cell cultures are widely used because of their relative simplicity, manipulability, and interpretability, and they allow for maximal flexibility in experimental control. While these models are fully compatible with manipulations of nutrient supply, genetic status, and tracer applications, they lack cell–cell interactions and 3D architectures. They have been valuable for both mechanistic and translational studies such as drug screening, with the caveat that these models do not always translate well to what occurs in vivo or clinically [109,110,111,112].

### 2.2. Xenograft and PDX Mouse Models

Mouse models with xenograft of established cancer cells or PD tissues (PDX) provide a more realistic TME with regard to 3D architectures and cell–cell interactions. However, these models cannot be used for studying immune-tumor cell interactions due to the use of immune compromised mice. Neither can they recapitulate proper patient tumor-stromal cell interactions, as xenograft models lack patient stromal cells to begin with and PDX models lose them after 2–3 generations [107,108]. Moreover, these models have no appropriate control tissue, limitations on experimental control, and complications in interpretability due to inter-organ interactions. Nevertheless, some PDX models have been shown to provide valuable preclinical information, such as mechanism of drug resistance [113] or responses [114,115].

### 2.3. 3D Spheroids and Organoids

An increasingly popular model is 3D cell culture that retains 3D architecture, cell–cell interactions and experimental control without the complexity of inter-organ interactions present in a whole organism. Three-dimensional cultures were first described by Bissel, who noted that breast cancer cells spontaneously formed acinar structures when grown on a supporting matrix [116,117,118]. Since then, 3D cultures and organoids have been developed for a wide range of cell types [49,108,111,112,119,120,121,122,123,124,125]. There are many types of support matrices now available, from the widely used Matrigel (a mouse sarcoma cell extracellular matrix) to other organic polymer supports such as alginates and hydrogels [126,127,128,129,130,131,132,133,134]. Three-dimensional cultures can also be induced without matrix support by concentrating “magnetized” cells via introduction of nano magnetic beads under a magnetic field [121,135]. Such close cellular contact allows the formation of cell–cell junctions and extracellular matrix (ECM) [109]. Indeed, concentration of cells by centrifugation can be sufficient to induce 3D structure formation on low adherence plates [136,137] or in scaffold-free hanging droplets [138,139]. It is practical to build complex 3D structures comprising multiple cell types as co-cultures either with established cell lines (spheroids) or PD cells (PDO) that correspond more closely to the organ or tumor tissues of interest. The PDO models [49] can show macroscopic functional responses [119,120,140] markedly different from those of the 2D counterparts and closer to the patient responses [121,133,134] due to their ability to emulate the 3D architectures and cell–cell interactions in patient’s TME.

Although genomics [141,142,143,144,145,146,147,148] and proteomics [149,150] approaches have been widely applied to studying spheroids and organoids, there have been comparatively few studies of metabolism in these models [35,108,124,151,152,153,154,155,156,157,158,159,160,161,162,163,164] and very few stable isotope tracers-based studies [35,121,124,162]. From the perspective of SIRM, not all 3D matrices are equal. First, Matrigel, a commonly used matrix, is an ECM derived from mouse sarcoma and is essentially an undefined medium that can vary in composition from batch to batch, leading to variable metabolic responses. Second, exchanging the growth medium in the matrix to the treatment medium is not trivial. Third, the matrix is difficult to remove fully from the embedded spheroids or organoids for harvesting, and the process is slow compared to the metabolic time scale. This can lead to undesirable changes in metabolite levels and difficulty in normalizing metabolite levels. For SIRM studies, fractional enrichments in the labeled metabolites are not influenced by the contamination of residual matrix, as they are internally normalized, but the slow speed of metabolic quenching remains a confounding factor for the biological interpretation.

An alternative to Matrigel is one of the many synthetic polymer supports, which are defined in composition and are batchwise reproducible, thereby eliminating batch artifacts and influence over protein-based normalization of metabolite content. However, the problem of medium exchange remains. It is also practical to use matrix-free magnetic-bead-based 3D culture method for metabolic studies [121]. In some systems, spheroids or organoids form without added matrix support, as the cells generate their own ECM [106,165,166]. In the latter two cases, control of the medium compositions and metabolic quenching will be straightforward, as the case for 2D cell culture studies.

Although spheroid or organoid assembly can emulate cell–cell interactions analogous to those found in tissues, cells in the assembly can assume different phenotypes depending on the assembly size and the nature of the matrix, with cells on the periphery showing different properties from those in the core [121,149]. To emulate the cellular heterogeneity of tissues, 3D co-cultures can be implemented, e.g., using mixtures of cancer cells plus immune cells, fibroblasts or endothelial cells [49,132]. The added complexity however makes it difficult to delineate the metabolic activity of each cell type, unless single cell analysis is carried out in situ. For metabolomic analysis, this remains challenging because of the low abundance of most metabolites in each cell and the difficulty of maintaining biochemical integrity and minimizing metabolite diffusion during cell isolation or in situ analysis [55]. For example, a metabolite present at 1 μM in a cell of 1 pL volume is only one attomole of material, which while achievable [167] is challenging to quantify even by the best mass spectrometers under ideal conditions, and is well beyond the capabilities of NMR [168] even with hyperpolarization [169]. To the best of our knowledge, single cell tracer studies have not been reported, presumably due to detectability issue. As isotopic enrichment increases the number of labeled species per metabolite to analyze, the detection sensitivity for each metabolite decreases. For example, with [U-^13^C]-glucose as the tracer, glutamate signal can be split into up to six different MS peaks each representing a ^13^C isotopologue species.

Before single cell SIRM is practically attainable, several complementary approaches can be employed to help resolve heterologous metabolic contribution from each cell type and metabolic interactions between cell types in 3D co-cultures or tissues. Such approaches are generally applicable without the severe limitation on the coverage of metabolic pathways afforded by single cell metabolomics. For example, if the influence of cancer cells on macrophages (Mφ), fibroblasts or T cells (and vice versa) were to be studied, one can first obtain under identical culture conditions metabolomic and RNAseq data from the pure spheroids of each cell type for comparison with the data acquired from the spheroid of mixed cell types at different cancer to stromal cell ratios. The mixed spheroids can also be subjected to microscopy including DSP for cell type-specific distribution of key molecular markers (proteins and mRNA) for phenotypes (e.g., apoptosis, proliferation) and metabolic pathways that are shown to be impacted from the bulk SIRM and RNAseq data. The cell-type-specific metabolic markers will help delineate each cell type’s contribution to the altered metabolic activity mapped by the bulk SIRM analysis. For example, elevated expression of glycolytic enzymes in Mφ but not in cancer cells in the co-culture point to macrophages’ contribution to enhanced glycolysis informed by the bulk SIRM data. Consequently, the impact of cancer cells on Mφ metabolism can be deduced by comparing Mφ metabolism in pure versus mixed 3D cultures.

To investigate the influence of diffusible substrates on target cell metabolism, one can treat, for example Mφ spheroids with cancer cell conditioned media (CM) and perform SIRM analysis for altered metabolic pathways. Alternatively, one can perform SIRM study on spheroids of each cell type separated by a membrane, which permits diffusion of small molecules but prevents heterologous cell–cell contact. Each spheroid culture can then be analyzed free of the other cell types. By comparing SIRM data thus obtained with those acquired from mixed co-cultures described above, one can resolve the influence of diffusible substrates from that of cell–cell contract on metabolic interactions between cell types.

We have begun to develop the above described approaches to investigate metabolic interactions between human lung cancer cells and Mφ in spheroid co-cultures. We have also introduced mSIRM [97,107,121,170] in our studies to maximize information retrieval from limited samples and to minimize batch artifacts. The following describes some preliminary findings that illustrate the value of these approaches.

## 3. Cancer Cell Conditioned Medium Has a Profound Effect on Human Mφ Metabolism and Effector Release

To determine whether diffusible compounds excreted from cancer cells impact human Mφ metabolism in response to polarization and an immune modulator β-glucan formulated as whole glucan particulates (WGP) we incubated Mφ in CM from A549 cells. WGP is a yeast-derived D-glucose polymer in linear β-1,3 linkages, which we have shown previously to repolarize mouse Mφ in 2D culture from the anti-inflammatory (M2) to the pro-inflammatory (M1) phenotypes while inducing M1-like metabolic responses [171]. The effect of polarization and WGP’s effect on human Mφ metabolism has not been studied, to the best of our knowledge.

Using ^13^C_6_-glucose (^13^C_6_-Glc) as a tracer, we performed a SIRM experiment on human Mφ differentiated for 6 days in DMEM medium containing 10% FBS, 0.2% glucose (Glc), and 50 ng/mL CSF1 (M0 medium) from a volunteer’s (UK96) peripheral blood monocytes (PBMC). On the 5^th^ day of differentiation, magnetic nanoparticles (Nanoshuttle, N3D) were loaded into the cells overnight, followed by reseeding of cells and spheroid formation under a strong magnetic field [121]. The spheroids were then incubated in M0 medium or polarized in M0 medium plus 100 ng/mL LPS + 20 ng/mL IFNγ to M1- or 20 ng/mL each IL4 + IL13 to M2-type Mφ for three days, followed by one day of culturing in M0 medium for the M0-, M1-, and M2-Mφ and in M0 medium plus 0.1 mg/mL WGP for the M2+WGP-Mφ in the presence of ^13^C_6_-Glc. The same polarization/^13^C_6_-Glc experiment was performed in parallel with the addition of A549 CM at 1:1 ratio to the polarization media. The CM medium was prepared by culturing A549 cells in M0 medium (with 0.1% Glc) for 24 h (1.16 million cells per 10 cm plate) and passing the medium through a 0.22 µM filter before use. The polar metabolites were extracted from cells and culture media respectively with 70% cold ethanol and 80% cold acetone. Due to the limited number of PBMC available from the volunteer, 200,000 cells were seeded each into two wells of a 96-well plate per treatment (n = 2), but the two replicate cell extracts were combined for the analysis by IC-UHR-FTMS, while the medium extracts for each well were analyzed separately by ^1^H NMR as described previously [121].

**Figure 2:**
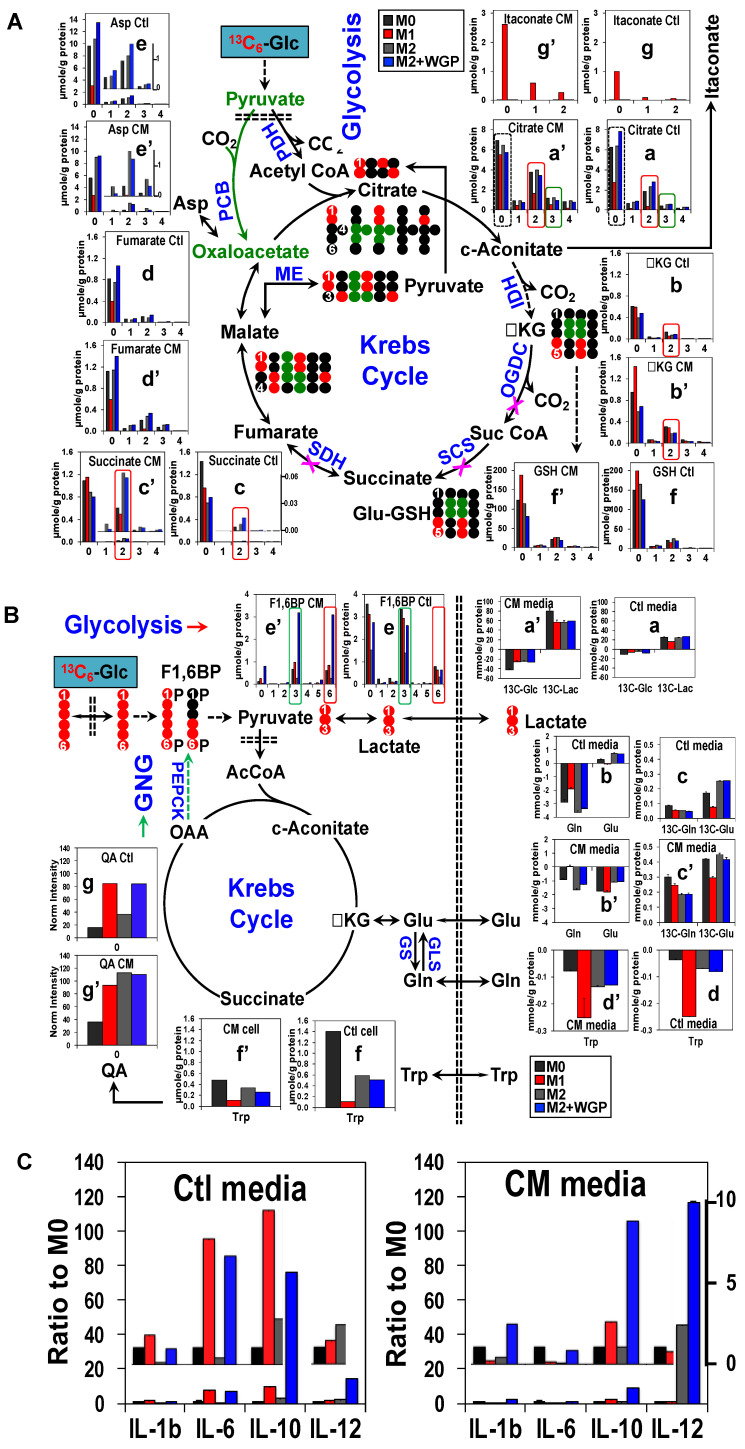
A549 cancer cell conditioned medium alters metabolic response of human macrophage (Mφ) spheroids to polarization and WGP as tracked by SIRM. The 3D Mφ cultures (n = 2) were prepared, polarized, and treated with ^13^C_6_-glucose (Glc) as described in the main text. The cell extracts were combined while the medium extracts remained separate for IC-UHR-FTMS analysis. ^12^C (●) and ^13^C (●,●) atom tracing through glycolysis, the Krebs cycle, gluconeogenesis (GNG), and Trp catabolism is shown to account for some of the labeled isotopologues of metabolites seen by IC-UHR-FT MS analysis. Red and green boxes in (**A**) denote labeled species produced respectively by glycolysis and the GNG pathway while black, red, and green boxes in (**B**) mark ^12^C, ^12^C_2_, and ^12^C_3_ isotopologues of metabolites. Numbers in X-axis refer to the number of ^13^C atoms. ∎: M0-Mφ; ∎: M1-Mφ; ∎: M2-Mφ; ∎: M2-Mφ+WGP. (**C**). Cytokines released into the culture media were measured using the Human Cytokine Magnetic 35-Plex Panel per vendor’s protocol (Invitrogen) and the level was ratioed to that of the M0 state. ∎: M0-Mφ; ∎: M1-Mφ; ∎: M2-Mφ; ∎: M2-Mφ+WGP. M0, M1 and M2 are three states of polarization of macrophages as described in the Methods.

Figure 2A shows the effect of the four treatments ± A549 CM on the uptake of nutrients and the release of metabolites into the medium along with the changes of related intracellular metabolites. In the absence of CM (Ctl), the uptake of ^13^C_6_-Glc (a) was somewhat enhanced in M1- (∎) and M2+WGP-Mφ (∎) compared with M2-Mφ (∎), which led to increased buildup of intracellular ^13^C_6_-fructose-1,6-bisphosphate (F1,6BP, e) but not the release of ^13^C-lactate (Lac, a) into the medium. The latter reflected little changes in the glycolytic capacity under different polarization treatments. We also saw enhanced buildup of ^13^C_3_-F1,6BP in M1-Mφ and M2+WGP-Mφ versus M2-Mφ, which could reflect increased gluconeogenic (GNG) capacity. Glutamine (Gln) uptake as well as the release of glutamate (Glu) (b) and ^13^C_6_-Glc-derived Glu (c) into the medium were lower in M1-Mφ than M2-Mφ and M2+WGP-Mφ. Glu and ^13^C-Glu are presumably exported for cystine uptake via the Xc- transporter system. We also saw newly synthesized Gln (^13^C-Gln, c) to be released into the medium but polarization treatments had little effect on this event. Tryptophan (Trp) uptake was enhanced with depletion of intracellular Trp in M1-Mφ versus M2- and M2+WGP-Mφ (d). This, together with the enhanced buildup of the downstream catabolite quinolinate (QA) in M1-Mφ, points to the activation of Trp catabolism but blockade of NAD^+^ synthesis from QA, which are consistent with the overexpression of indoleamine 2,3-dioxygenase 1 (IDO1) [172] and the blockade of quinolinate phosphoribosyltransferase (QPRT) [173] in inflammatory human Mφ. QA buildup also occurred in M2+WGP-Mφ but without the enhanced Trp uptake and depletion of intracellular Trp. When CM was present, we saw enhanced ^13^C_6_-Glc uptake and ^13^C-Lac release (a’), which was independent of the polarization/WGP treatments. In contrast, CM reduced Gln uptake while reversing Glu release into the medium, particularly for M1-Mφ (b’). However, CM enhanced the release of newly synthesized Gln and Glu into the medium (c’), relatively more so for M1-Mφthan for M2-Mφand M2+WGP-Mφ. Moreover, CM enhanced Trp uptake (d’) and QA buildup (g’) with reduced Trp accumulation (f’) in M2-Mφ and M2+WGP-Mφ but had little effect on the corresponding events in M1-Mφ.

To see the treatment effect on the Krebs cycle, we examine the ^13^C labeling patterns of Krebs cycle metabolites in human Mφ in response to polarization ± CM treatments by IC-UHR-FTMS analysis. Figure 2B shows reduced levels of the ^13^C_2_- and ^13^C_3_-isotologues of citrate (**a**), succinate (**c**), fumarate (**d**), and Asp (**e**) in M1- versus M2- and M2+WGP-Mφ, which suggests decreased capacity of the pyruvate dehydrogenase (PDH) and pyruvate carboxylase (PCB)-initiated Krebs cycle, respectively [91]. This is consistent with the compromised Krebs cycle observed in mouse M1-Mφ due to the two breaks at the isocitrate dehydrogenase (IDH) and succinate dehydrogenase (SDH) steps [174,175,176]. We observed succinate buildup and fumarate depletion expected from the SDH break but did not see citrate buildup and αketoglutarate (αKG, **b**) depletion expected from the IDH block in the Krebs cycle. These changes applied only to the ^12^C (unlabeled) isotopologues, which indicate that they are not derived from ^13^C_6_-Glc metabolism. Instead, ^13^C_6_-Glc-related buildup of ^13^C_2_-αKG (b) and depletion of ^13^C_2_-succinate (c) in M1-Mφ versus M2- and M2+WGP-Mφ were evident, which is likely due to a block at the oxoglutarate dehydrogenase (OGDH) and/or succinyl CoA synthetase (SCS) step. Despite the attenuated Krebs cycle capacity, ^13^C labeling and/or the levels of the byproducts, itaconate (g) and glutathione (GSH, f), were elevated in M1-Mφ. Itaconate is known to be elicited by LPS in inflammatory Mφ to inhibit microbial growth [177], but its excess buildup was also shown to be anti-inflammatory [178,179]. GSH has been shown to stimulate pro-inflammatory activity while relieving oxidative stress in RAW 264.7 macrophages [180]. Moreover, we saw multiple CM-dependent changes in the Krebs cycle, notably the enhanced buildup of both unlabeled and ^13^C-itaconate (g’) as well as the less blocked Krebs cycle in M1-Mφ as evidenced by the less depletion of ^13^C labeled Krebs cycle products (a’, c’, and d’) relative to M2-Mφ. These changes could signify a shift towards the M2-like phenotype, which is consistent with the CM-induced decrease in the release of pro-inflammatory cytokines IL-1β and IL-6 by M1-Mφ as shown in Figure 2C. Also shown was the stimulation of IL-1β and IL-6 release into the M2-Mφ media by WGP, which points to a shift from M2 to an M1-like phenotype. Further consistent with the M2 to M1 switch was the enhanced release of IL-10 into the M2-Mφ media by WGP. IL-1β, IL-10, and IL-12 were previously shown to be markers of human M1-Mφ in 2D cultures [172]. Here we showed this to be the case for IL-1β and IL-10 but not for IL-12 in M1-Mφ as spheroid cultures. IL-12 release by M2-Mφ was stimulated by WGP or CM and further enhanced by CM+WGP. Although known as a pro-inflammatory cytokine, IL-12 can also have an anti-inflammatory role during secondary immune responses [181]. Further studies are needed to see if how IL-12 alters immune functions in response to WGP and/or CM treatments.

It should be noted that M1 and M2 designations do not fully represent the complexity of human macrophage phenotypes [182], nor can they fully describe the WGP effect on Mφ polarization in spheroid cultures. Based on the above data, WGP induced a mixed phenotype in human M2-Mφ spheroids, with some M1-like features (e.g., QA buildup; enhanced IL-1β/IL-6 release) while retaining a large part of the M2 properties (e.g., enhanced Gln uptake/Krebs cycle activity).

## 4. Co-Culturing of Cancer Cell with Mφ Alters Metabolic Response of Human M2-Mφ Spheroids to WGP

**Figure 3:**
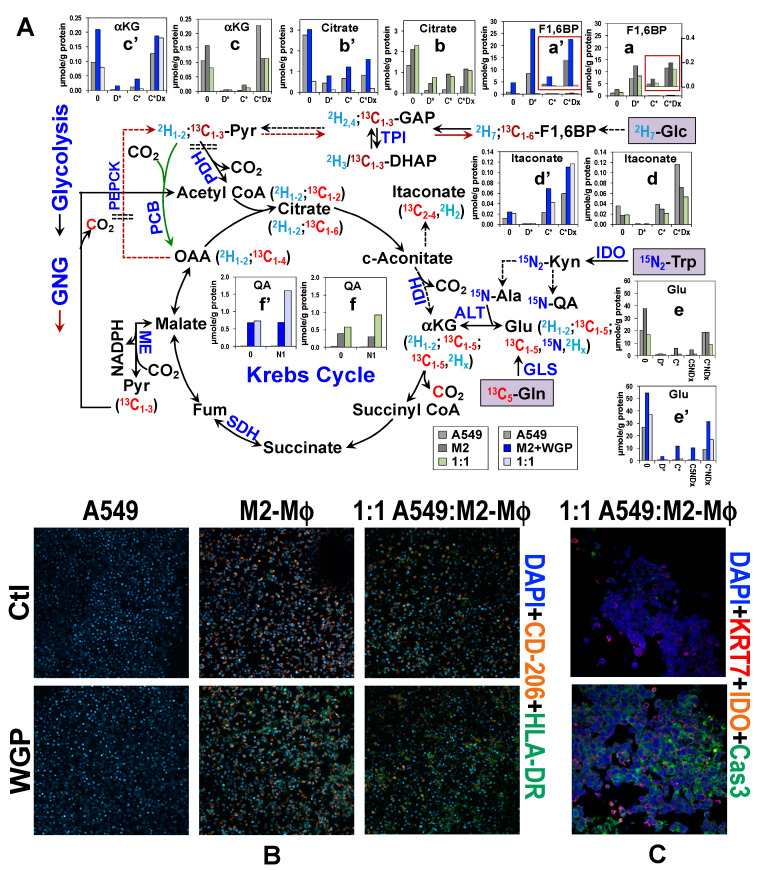
A549 cancer cell co-culturing alters metabolic and immune marker responses of human M2-Mφ spheroids to WGP as tracked by mSIRM. The 3D single or co-cultures (1:1 A549:Mφ) (n = 2) in (**A**) were treated with IL-4+IL-13 for 3 d ± WGP as in Figure 2 except with 2H7-Glc+13C5-Gln+15N2-Trp as tracers in the last 24 h. The cell extracts were combined for IC-UHR-FTMS analysis. 2H/13C/15N atom tracing through glycolysis, the Krebs cycle, GNG, and Trp catabolism is shown to account for some of the labeled isotopologues of metabolites seen by IC-UHR-FTICRMS analysis. Red boxes denote 13C labeled fructose-1,6-bisphosphate (F1,6BP) produced by the GNG pathway. ∎: A549; ∎: M2-Mφ; ∎: A549:M2-Mφ 1:1; ∎: M2+WGP; ∎: A549:M2-Mφ 1:1+WGP; D*: total 2H; C*: total 13C; N: 15N; Cx: 13Cx; Dx: 2H0-x; GAP: glyceraldehyde-3-phosphate; DHAP: dihydroxyacetone-3-phpsphate; Pyr: pyruvate; αKG: αketoglutarate; Fum: fumarate; OAA: oxaloacetate; Kyn: kynurenine; QA: quinolinate; TPI: triosephosphate isomerase; PDH: pyruvate dehydrogenase; PCB: pyruvate carboxylase; ALT: alanine transaminase; SDH: succinate dehydrogenase; ME: malic enzyme; PEPCK: phosphoenolpyruvate carboxykinase; IDO: indoleamine 2,3-dioxygenase; GLS: glutaminase. In (**B**), treated mono- and co-cultures were stained as live cells for M2 (CD-206), M1 (HLA-DR), and nuclear (DAPI) markers while in (**C**), cells were fixed in 4% paraformaldehyde before staining for cancer cell (KRT7), M1 (IDO1), and apoptotic caspase 3 (Cas3) markers.

As described above and in Table 1, direct cell–cell interactions and altered architecture of tumors can impact cancer and stromal cell metabolism. We mixed A549 cells with differentiated Mφ from patient UK96 in a 1:1 ratio as spheroids and subjected the co-cultures in parallel with spheroids generated from isolated A549 or Mφ cells to the same polarization and WGP treatment as described above except for employing a triple tracer cocktail 0.2% ^2^H_7_-Glc + 2 mM ^13^C_5_-Gln + 78 µM ^15^N_2_-Trp for 24 h. The incorporation of ^2^H, ^13^C, and/ or ^15^N into various metabolites was resolved and quantified by IC-UHR-FTMS [107,170,183,184]. Figure 3A illustrates the tracing of ^2^H_7_-Glc via glycolysis and the Krebs cycle, of ^13^C_5_-Gln via glutaminolysis, the Krebs cycle, and GNG, and of ^15^N_2_-Trp via the kynurenine (KYN) pathway [173,185] in the pure cultures and co-culture under M2 polarization ± WGP treatment. We found that M2-Mφ spheroids (∎) had a higher capacity for incorporating ^2^H (D), ^13^C, and/or ^15^N into glycolytic (e.g., F1,6BP, a), Krebs cycle (e.g., citrate, b; αKG, c; and Glu, e), and Trp metabolites (e.g., QA, f) than A549 spheroids (∎). The production of ^13^C-F1,6BP ^13^C_5_-Gln from signified GNG activity (→). All of these capacities were further enhanced by the WGP treatment (∎, a’-c’ and e’). WGP also increased the buildup of ^13^C-itaconate in M2-Mφ but it had an opposite effect in A549 spheroids (d’). The WGP effect on the Krebs cycle and GNG in M2-Mφ spheroids recapitulated those traced by ^13^C_6_-Glc in Figure 2 while the enhanced buildup of ^15^N-QA in WGP treated M2-Mφ spheroids verified altered Trp catabolism reasoned in the ^13^C_6_-Glc study.

Co-culturing of A549 and M2-Mφ (∎) did not have a notable effect on glycolysis (**a**) but enhanced the buildup of ^13^C and/or D-labeled (C*D_x_, b) citrate and ^15^N-QA (f) while depleting C*D_x_-αKG (c), ^13^C, ^15^N, and/or D-labeled (C*ND_x_) Glu (e), and C*D_x_-itaconate (d) relative to the A549 monoculture. These metabolic interactions were significantly modified by the WGP treatment (∎). WGP greatly suppressed ^13^C and/or D incorporation into F1,6BP (a’), citrate (b’), while having an opposite effect on that into αKG (c’)/ itaconate (d’), and ^15^N incorporation into QA (f’) in the co-culture (Figure 3A). These effects were either opposite (for F1,6BP, Figure 2A) or absent in the CM+WGP-treated M2-Mφ (for QA, Figure 2A; citrate, αKG, and itaconate, Figure 2B), which points to the importance of cell–cell contact in mediating these effects. Such co-culturing+WGP effect on citrate and αKG was akin to the CM-induced responses in M1-Mφ (a’ versus b’, Figure 2B), which could reflect a break at the OGDC or SCS step. This, together with the enhanced buildup of itaconate and QA suggests that WGP retained its ability to repolarize the M2 to M1 phenotypes in the co-culture but also enhanced further the M2 phenotype based on the F1,6BP response. The mixed M1- and M2-type metabolic responses induced by WGP in both mono- and co-cultures of M2-Mφ were consistent with the expression of both M1 (HLA-DR) and M2 (CD-206) markers (Figure 3B). WGP treatment also induced the expression of another M1 marker (IDO1) and apoptotic marker caspase 3 (Figure 3C), which implicates killing action via M1 repolarization.

The WGP’s action on the metabolic and immune responses in the 3D cancer cell-Mφ co-culture can be related to those of the PD OTC of tumor tissues [186] to help resolve the contribution of cancer cells or tumor-associated Mφ (TAM) to the overall responses. For example, the WGP-induced buildup of glucose-derived F1,6BP in the responsive tumor tissue (Figure 2B in Fan et al. [186]) could have originated from Mφ not in direct contact with cancer cells (Figure 2A), as this response was not evident in the mono-cultures of cancer cells and M2-Mφ or in their co-culture with cell contact (Figure 3A). By a similar reasoning, the WGP-induced accumulation of unlabeled itaconate in the responsive tumor tissue (Figure 2L in [186]) was likely a result of enhanced Gln, instead of Glc, metabolism in cancer cells/Mφ alone and/or cancer cells-TAM that are in direct interactions (Figure 3A). This interpretation was afforded with very limited samples using the mSIRM approach, where the fate of Glc and Gln were tracked simultaneously and more robustly than the single tracer-based SIRM approach.

## 5. Concluding Remarks and Future Directions

To achieve systems biochemical understanding of tissue heterogeneity at the cellular level, it would be ideal to perform SIRM, proteomic, and genomic analyses at the individual cell level in live tissues in situ [108,159,187,188]. However, this is not yet technically feasible. Alternative strategies that combine techniques at different scales should therefore be considered.

Subcellular imaging of cells and tissues has long been feasible with epi and confocal fluorescence microscopy. Recent advancement in super resolution imaging can achieve image resolution of 50 nm or less [189,190,191,192,193]. In addition, free versus bound concentrations of metabolites can be discriminated in cells by fluorescence lifetime imaging microscopy (FLIM) [158,194,195]. However, microscopy-based metabolic imaging requires the use of fluorescent probes such as NBD-glucose for measuring glucose uptake [58,59,60], with the exception of a few metabolites that have intrinsic fluorescence (e.g., FADH2, NAD(P)H) [196]. In addition, this approach cannot resolve stable isotopes and is currently low in metabolic coverage. More recently, high resolution confocal Raman imaging has become practical with single cell resolution. As the Raman effect depends on bond vibrations, signals are sensitive to isotopic substitutions, especially for D replacement of H [197,198,199]. Although not directly useful for metabolic imaging, the emerging DSP technique can be used to measure the expression of metabolic genes and proteins in situ at single cell type resolution. Such information can help resolve the contribution of single cell types to the metabolic activity measured by SIRM and protein expression analyzed by RPPA [107,200] in tissues with no spatial discrimination.

NMR-based imaging (NMRI) does not require optical probes, has greater depth than optical imaging, is capable of wider metabolite coverage with stable isotope resolution [201,202,203,204,205,206], and is fully compatible with in vivo real-time measurements including metabolic rates (e.g., unidirectional rate of ATP synthesis [207,208]). However, its utility is limited by much poorer resolution (regional level) and lower sensitivity. The sensitivity of stable isotope-resolved MRI can be substantially improved by the use of hyperpolarized substrates [209,210,211], post-acquisition processing [11], and proton detection [201,212,213,214] but still at relatively low spatial resolution and metabolic coverage (typically < 10 metabolites).

MALDI-based mass spectrometric imaging can achieve high resolution in tissue, at either regional resolution comparable to DSP or at the single cell (1–10 μm) resolution with new developments [215,216,217,218]. These technologies are particularly valuable for resolving spatial heterogeneities of immobile lipids, proteins, and glycoproteins, such as glycosylated surface protein and extracellular matrix peptides [219,220].

DSP coupled with SIRM approach can be applied to spheroid and organoids for resolving their metabolic heterogeneities. As we have shown, it is also feasible to use SIRM to profile metabolic activities in mono- and co-spheroid cultures of different cell types to learn about their metabolic distinction and interactions, which can in turn help determine individual cell types’ contribution to bulk tissue metabolism. We have demonstrated that the SIRM profile of human macrophages is altered by human lung A549 cancer cell conditioned media (which contains excreted diffusible compounds), and by coculturing with A549 cell. However, the profiles of CM versus co-culture are not identical, indicating that the cell–cell contacts induce altered metabolism differently from the diffusible substrates. This strategy is greatly facilitated by the new mSIRM technology, which we have demonstrated as a preliminary investigation. Future organoid and matched tissue studies that integrate mSIRM with metabolic imaging, DSP, as well as single-cell ‘omics, should achieve global mapping of metabolic activity at the single cell levels before single-cell SIRM becomes a reality. New informatics and statistical analyses that enable robust and efficient information retrieval as well as integrated data interpretation from such studies will also need to be developed.

## 6. Materials and Methods 

Lung tissues were collected from consented patients at the operating room within five minutes of surgical resection and placed in DMEM media in accordance with HIPAA regulations. All tissue experiments were carried out via a protocol approved by the University of Kentucky Institutional Review Board (IRB) (IRB 14-0288-F6A). The pair of tissues were thinly sliced to <1 mm thickness as previously described [221,222]. Tissues for the tumor and adjacent healthy lung were preserved in formalin and embedded in paraffin for pathological analyses

### 6.1. H&E Staining

Thin paraffin-embedded sections of tissues were H&E stained as per standard protocol. A variety of cell types can be identified from size and shape in the cancer tissue, including transformed epithelia, plasma cells, infiltrating lymphocytes and macrophages, as shown in Figure 1A.

### 6.2. DSP (Nanostring)

FFPE tissue slices from human NSCLC were cut to 5 μm and sent to NanoString (Seattle, WA, USA) for staining for different cellular markers, including the nuclei (for counting cells), and fluorescent antibodies to keratin (pan cancer markers), CD8 (T cell marker), and CD68 (macrophage marker).

Protein markers in regions of interest of the FFPE tissue sections were quantified by counting the cleaved oligos using Nanostring’s nCounter. The oligo-conjugated antibodies were prepared and validated by Nanostring. ROIs in fixed tissues were selected based on desired markers for cell nuclei and markers for T-cells, macrophages, and in consultation with a pathologist (Dr. T. Bocklage) before automated analysis on Nanostring’s GeoMx system and nCounter.

### 6.3. Monocytes Isolation, Differentiation, and Polarization

Monocytes were isolated from whole human peripheral blood of a male (UK96) and a female (UK94) donors (both >60 years of age) using the RosetteSep™ human monocyte enrichment cocktail kit (StemCell, Cambridge, MA). Monocytes were differentiated in 6-well plates (Nunclon, ThermoFisher Scientific, USA) for six days in monocytes differentiation medium (MDM) containing DMEM, 10% FBS, 10 mM glucose, 2 mM Gln, 1X Anti-anti, and 50 ng/mL CSF-1 at 37 °C/5% CO_2_ at a density of 3–5 × 10^6^ cells per well before polarization. Cells were maintained in MDM (M0) or polarized to either M1-like with 100 ng/mL LPS + 20 ng/mL IFNγ or to M2-like subtype with 20 ng/mL each IL-4 + IL-13 for 2 to 3 days. 

### 6.4. SIRM of Macrophage Spheroids and A549-Macrophage Organoid Cultures

Human macrophages were isolated as described above were induced to form spheroids in 6-well plates in organoid medium and were treated with 10 mM ^13^C_6_-glucose for 24 h before harvesting and immediate extraction for polar, non-polar and protein fractions using our CH_3_CN/H_2_O/CHCl_3_ solvent partitioning method [223,224]. After lyophilization and reconstitution, polar metabolites were identified and quantified by IC-FTMS and NMR according to our untargeted workflow that can resolve multiple tracer atoms (e.g., ^2^H, ^13^C, and ^15^N) in the same metabolite [91,92,170,225,226,227,228]. For co-cultures, macrophages were mixed with an equal number of A549 cells and induced to form spheroids and incubated for 24 h with medium containing 10 mM ^2^H_7_-glucose + 70 μM ^15^N_2_ tryptophan + 2 mM ^13^C_5_ glutamine. The organoids were harvested and extracted for analysis as described for the macrophages.

## Figures and Tables

**Table 1:** Tumor tissue heterogeneity. Scale refers to the size of the unit, from individual cells (local) to groups of cells or tissue (regional) to global (organism).

Scale	Heterogeneity	Examples	Refs.
Global	Cell types	Normal and transformed epithelia, fibroblasts, endothelia, and resident and infiltrating immune cells.	Figure 1; [14,15]
Regional	Cancer cellularity	< 10 - > 90% of total cells	[16,17]
Regional	vascularity	Restricted flow -> local hypoxia, nutrient deprivation, waste buildup; gradients in IF impacts on cell gene expression.	[18,19,20,21]
Regional/local	Disrupted ECM and tissue organization	Altered cell interactions: impacts on cell gene expression.	[21,22]
Regional/local	Cell–cell interactions	Direct cell contacts versus interaction via diffusible molecules: altered behavior of T cells, macrophage polarization (TAMs), and fibroblast activity (CAFs).	[23,24]
Global	Cell–cell interactions	Tissue polarity impacts cell function by position - cells or groups of cells have different metabolic activities according to position, and different cell types have different metabolic activities. The “intrinsic” metabolic phenotypes of cells are greatly influenced by interactions within heterogeneous tissues.	[25,26]
Regional/local	Cell distribution	Cell distribution is highly heterogeneous (clumps and voids—regional versus cellular heterogeneity).	Figure 1; [27,28]
Local	Cells	Cells within tumors may have different expression patterns as well as different genome alterations. Expression patterns may vary in part from environmental influences on epigenetics (chromatin structure).	[24,29,30]
Regional	Necrosis	Heterogeneous because of variable necrosis in different regions of the tumor	[31]
Organ	Tissue-dependent tumors; subtypes	Tumors of the same tissue origin are heterogeneous—subtypes (adeno versus squamous versus NET etc.) that are characterized by different functional properties. Some subtypes can interconvert (cf. lung adenosquamous phenotype). Cancer cells can also undergo EMT. Cells may de-differentiate or even trans differentiate.	[32,33,34,35,36,37,38]
Local	Cell structure	Cells are compartmented and heterogeneous.	[39]

## Abbreviations

CAF cancer associated fibroblast; CM Conditioned Medium; CSF1 colony stimulating factor 1; DSP digital spatial profiling; ECM extracellular matrix; EMT epithelial mesenchyme transition; FFPE formalin fixed paraffin embedded; Glc D-glucose; ^13^C_6_-Glc glucose enriched with ^13^C at all six carbon positions; H&E hematoxylin and eosin; IC-UHR-FT MS ion chromatography-ultrahigh resolution mass spectrometry; MALDI matrix assisted laser desorption ionization; Mφ macrophages; NSCLC non-small cell lung cancer; NSG Nod Scid Gamma; PDX patient-derived xenograft; RPPA reverse phase protein array; scRNAseq single cell RNA sequencing; SIRM Stable Isotope Resolved Metabolomics; TAM tissue associated macrophage; TME tumor microenvironment; WGP whole glucan particles; UHR-FTMS ultra high resolution Fourier transform mass spectrometry.
